# Do Adults Who Practice Aerobic Physical Activities Have Different Influencing Factors for Depression: A Secondary Data Analysis

**DOI:** 10.3390/ijerph19106142

**Published:** 2022-05-18

**Authors:** Sun Ae Kim, Youngshin Song, Myoungjin Kwon

**Affiliations:** 1 Department of Nursing, Korea National University of Transportation, Jeungpyeong-gun 27909, Korea; sakim@ut.ac.kr; 2 College of Nursing, Chungnam National University, Daejeon 35015, Korea; 3 Department of Nursing, Daejeon University, Daejeon 34520, Korea

**Keywords:** depression, adults, aerobic, activity

## Abstract

Practice in aerobic activities can positively impact depression. This study aimed to identify differences between adults who do and do not practice aerobic activities in terms of general, physical, and psychological factors that influence depression. This study comprised a secondary analysis of data from the 6th (year 2) and 7th (years 1, 3) editions of the Korea National Health and Nutrition Examination Survey. Data from 12,891 adults were analyzed, of whom 7148 reported no practice in aerobic physical activities and 5743 reported practicing such activities. Data were analyzed using SPSS 25. Among those who did not perform aerobic activities, gender, family income, marital status, obesity, frequency of drinking, subjective health, subjective body weight, and stress were found to influence the level of depression. For those who practiced aerobic activities, gender, age, family income, education level, marital status, blood pressure, hypercholesterolemia status, frequency of drinking, subjective health, subjective body weight, and stress influenced the level of depression. This study found that the factors affecting depressive symptoms differ depending on whether individuals practice aerobic activities. Thus, to reduce depressive symptoms among adults, it is necessary to consider their level of physical activity and target the influencing factors associated with this level.

## 1. Introduction

Today, there are approximately 280 million cases of depression worldwide [1]. Depression can have numerous negative health effects, including causing sleep pattern disturbance, sadness, loss of pleasure, loss of interest, irritability, guilty feeling, agitation [2], social isolation [3], poor quality of life [4], and can also increase the risk of mortality [5] and physical illness [6]; in particular, depression is a risk factor for suicidal ideation [7]. Thus, addressing depression is especially important for countries such as South Korea, which has the highest suicide rate among the OECD countries and Development countries [7]. Moreover, depression is the most prevalent mental illness among adults in Korea. Factors influencing depression in adults include general characteristics such as gender, age, marital status, education, and socioeconomic status; physical factors such as chronic diseases (e.g., hypertension and diabetes), lifestyle habits (e.g., smoking and drinking), and practice in physical activity; and psychological factors such as stress level and subjective physical condition [8,9,10,11]. Practice in aerobic activities has been found to have positive effects on emotional and physical health, as it can improve stress and mood states associated with depression and reduce the incidence of depressive episodes [12,13]. In order to prevent and control chronic diseases, WHO has emphasized the importance of physical activity and presented guidelines for different types of exercise. In other words, guidelines for muscle strengthening exercises as anaerobic activity and aerobic physical activity were presented [14]. This indicates that both types of physical activities are important in both physical and mental chronic diseases [15]. However, few studies have examined whether the influencing factors for depression differ between adults with different levels of practice in aerobic activity.

The present study sought to identify differences between adults who do and do not practice aerobic activity with respect to the general, physical, and psychological factors that influence depression. Regarding the relationship between aerobic physical activity and depression, a study confirmed the effect on depression when aerobic physical activity was applied as an intervention [16]. Additionally, a study confirmed the relationship between anaerobic physical activity and depression in American adults [17]. That is, they were different studies that independently confirmed the factors affecting aerobic and anerobic physical activity. Another study identified more effective types of exercise by classifying levels of physical activity and identifying factors affecting depression [18]. However, there have been no studies identifying the influencing factors of depression by classifying participants based on whether they did or did not perform aerobic physical activity in daily life.

In a previous study that identified risk factors for depression by age, the risk of depression for individuals in their 30s and 50s was significantly higher than those in their 20s [19]. Another study reported that depression gradually increased from the 40s [20], and these results confirmed that depression, which started to increase around the 30s, continued to increase after the 40s and 50s. Thus, in this study, it is necessary to identify risk factors for depression in adults over 30s. Therefore, the purpose of this study is to identify the influencing factors of depression by differentiating those who practice aerobic physical activity and those who do not.

## 2. Materials and Methods

### 2.1. Participants and Procedure

This study used data from the second year (2014) of the 6th Korea National Health and Nutrition Examination Survey (KNHANES) and from the 1st and 3rd years (2016, 2018) of the 7th KNHANES. The KNHANES is a nationwide health and nutrition examination survey conducted by the Korean Ministry of Health and Welfare and the Korea Centers for Disease Control and Prevention (KCDC), with the aim of producing representative and reliable statistics on national and municipal scales regarding the health status, health behavior, and nutrition status of the Korean people. Sampling bias was controlled through sampling guidelines in the KCDC to which clustered, multistage, stratified, and probability sampling was applied [21].

Prior to data collection, informed consent was obtained from all study participants, and data collection was performed by the KCDC in compliance with research ethics guidelines [21]. The data can be used for research purposes alone, can only be accessed in accordance with the KCDC guidelines, and do not contain information that can identify individual participants.

**Figure 1:**
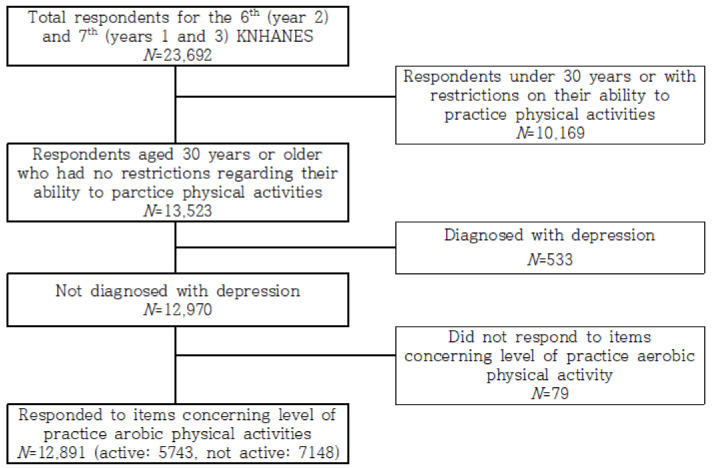
Participant selection process.

Among all participants (*n* = 23,692) included in the years targeted for this study, 12,891 were aged 30 or older, did not have any restrictions regarding their ability to practice in physical activity, had never been diagnosed with depression, and had provided details regarding their practice in aerobic physical activity (of these, 7148 adults said they did not practice in any physical activity and 5743 said they did; Figure 1).

### 2.2. Measures

#### 2.2.1. Sociodemographic Factors

Age was classified as 30–49, 50–64, and 65 years, family income was classified as upper, middle, and lower. Education level was categorized as middle school or lower, high school, and college graduate or higher, and the number of household members was categorized as one, two, and three or more. Furthermore, housing type was classified as detached house, apartment, and other, while marital status was classified as living with spouse and other (separated from spouse, bereavement, or divorce).

#### 2.2.2. Physical Factors

Blood pressure was categorized as normal (no high blood pressure and no prehypertension; systolic blood pressure less than 120 mmHg and diastolic blood pressure less than 80 mmHg), prehypertension (no high blood pressure; systolic blood pressure 120 mmHg or more, but less than 140 mmHg, diastolic blood pressure 80 mmHg or more, but less than 90 mmHg), and high blood pressure (systolic blood pressure above 140 mmHg or diastolic blood pressure above 90 mmHg), respectively. Body weight status was classified as low weight (body mass index [BMI] below 18.5 kg/m^2^), normal (BMI between 18.5 kg/m^2^ and 25 kg/m^2^), and obese (BMI above 25 kg/m^2^), respectively.

Diabetes status was categorized as normal (no diabetes and no impaired fasting glucose; fasting blood sugar less than 100 mg/dL), impaired fasting glucose (no diabetes; fasting blood sugar 100 mg/dL or more, but less than 126 mg/dL), and diabetes (fasting blood sugar over 126 mg/dL, medical diagnosis of diabetes, taking a hypoglycemic agent, or taking insulin injections), respectively. Hypercholesterolemia status was classified as hypercholesterolemia (having a total cholesterol level of 240 mg/dL or higher and/or taking cholesterol-lowering agents) or no hypercholesterolemia (total cholesterol level lower than 240 mg/dL and not taking cholesterol-lowering agents), respectively. Weight-change status over the previous year was categorized as increased, decreased, or no change, respectively, and weight-control status over the previous year was categorized as “made efforts to reduce weight”, “made efforts to increase weight”, “made efforts to maintain weight”, and “made no efforts regarding weight” respectively. Frequency of drinking over the previous year was classified as less than once a month, 2–4 times a month, and more than twice a week, respectively, and alcohol consumption per session was classified as 1–2 glasses, 3–6 glasses, and more than 7 glasses, respectively (regardless of the type of alcohol).

Regular practice in aerobic activities in daily life was defined as at least two hours and 30 min of moderate-intensity physical activity a week or one hour and 15 min of high-intensity physical activity a week; individuals who mixed high- and moderate-intensity activities were also included (e.g., high-intensity for one minute and moderate-intensity for two minutes).

#### 2.2.3. Psychological Factors

Subjective health was categorized as good, normal, and bad, respectively, while subjective body type recognition was categorized as thin, normal, and obese, respectively. Furthermore, perceived stress was categorized as low and high, respectively. Depression was measured using the Patient Health Questionnaire-9 (PHQ-9) developed by Spitzer, Kroenke, and Williams [22]. The PHQ-9 contains nine items, each of which is answered using a four-point Likert scale. The total score ranged from 0 to 27; the higher the score, the more severe the depression.

### 2.3. Statistical Analysis

After generating a planning file for the complex sample, the data were analyzed using weighting, and the significance level was set at 0.05. Analysis was performed using IBM SPSS Statistics for Windows, version 25.0 (IBM Corp., Armonk, NY, USA).

Missing data were processed in accordance with the KCDC’s statistical guidelines for the use of data from the KNHANES [21]. General characteristics, physical factors, and psychological factors were analyzed using frequencies and percentages. Frequencies were based on actual measurements, while percentages were determined using weight-based values. Differences between the aerobic physical activity group and no aerobic physical activity group regarding general characteristics, physical factors, and psychological factors were analyzed using a χ^2^-test, and factors influencing depression were analyzed using linear regression analysis.

## 3. Results

### 3.1. Participants’ Sociodemographic Characteristics

Significant differences were found between the no aerobic physical activity and aerobic physical activity groups for all general characteristic variables analyzed (Table 1). Those who practiced aerobic activities were significantly more likely to be men, aged 30–49 years, have a high family income, have college or higher education level, have a household of three or more members, live in an apartment, and to be living with one’s spouse (*p* < 0.001).

### 3.2. Participants’ Health-Related Characteristics

Comparing the aerobic physical activity group with the no aerobic physical activity group in terms of physical characteristics showed significant differences regarding blood pressure, body weight, diabetes status, hypercholesterolemia status, weight control, drinking status, frequency of drinking, and smoking status (Table 2). When compared to the no aerobic physical activity group, the aerobic physical activity group featured fewer individuals with hypertension, diabetes, and hypercholesterolemia, and had a lower average body weight as well. Further, the aerobic physical activity group made more efforts to lose weight, included more individuals who drank alcohol, and had a higher average for frequency of drinking. However, it contained fewer smokers (*p* < 0.05).

Significant intergroup differences were observed for all psychological characteristics. Those who practiced aerobic physical activities perceived themselves to be subjectively healthier when compared to those who did not practice aerobic physical exercise; the members of the former group also perceived their body type to be more obese and to have less stress (*p* = 0.040). Moreover, the aerobic physical activity group showed a lower prevalence of depression (*p* = 0.022).

### 3.3. Influencing Factors for Depression Depending on Aerobic Physical Activity Status

Table 3 presents the results of the factors associated with depression. For the no aerobic physical activity group, gender, age, family income, marital status, blood pressure, body weight, frequency of drinking, subjective health, subjective body weight, and stress had a significant influence on level of depression, with an explanatory power of 21.3% (*p* < 0.001). Being male (when compared to female), being aged 50–64 years (compared to being aged 65 years), having a higher family income, living with spouse, having a low frequency of drinking, having better subjective health, having normal or thin subjective body weight (when compared to obese), and having low perceived stress were associated with lower levels of depression. Meanwhile, having normal blood pressure (when compared to hypertension) and being underweight or normal weight (when compared to being obese) were associated with higher levels of depression.

For the aerobic physical activity group, gender, age, family income, education level, housing type, marital status, blood pressure, body weight, hypercholesterolemia status, frequency of drinking, subjective health, subjective body weight, and perceived stress had a significant influence on the level of depression, with an explanatory power of 24.6% (*p* < 0.001). Being male (when compared to being female), having higher family income, living with spouse, having a low frequency of drinking, having better subjective health, having normal or thin subjective body weight, and having low perceived stress were associated with lower levels of depression. Meanwhile, being aged 30–49 (when compared to being aged 65 or over), having lower education level, living in a detached house, having normal blood pressure (when compared to hypertension), having normal body weight (when compared to obese), and not having hypercholesterolemia (when compared to having hypercholesterolemia) were associated with higher levels of depression.

## 4. Discussion

This study sought to identify differences between adults who do and do not practice aerobic physical activity with respect to the general, physical, and psychological factors that influence depression.

In both groups, gender, family income, marital status, frequency of drinking, subjective health, subjective body weight, and stress were influencing factors, while the effects of body weight, age, education level, blood pressure, and hypercholesterolemia status were found to differ across the two groups.

Regarding our finding that females are more likely to experience depression than males, a previous study [23] similarly reported that women are 1.5–2 times more likely to experience major depressive disorder in their lifetime than men. Further, according to the 2016 Epidemiological Survey of Mental Illness conducted by Korea’s Ministry of Health and Welfare, the life prevalence of depression (major depressive disorder) is 3.1% for men and 6.9% for women; that is, more than twice as high for women than men [24]. Women generally have lower education levels and lower economic levels than men (due to their greater engagement in part-time work and exposure to unfavorable working conditions) [25,26]. In addition to these difficulties, women usually show greater concern about the well-being of their families and people in their state, and feel a need to care for others, which causes a psychological burden that increases their vulnerability to depression [27]. Thus, this may explain the greater prevalence of depression among women than men.

In a previous study [28], depression was found to be higher in groups with “bottom” incomes than in those with higher incomes, and to be lower among individuals with spouses than among those without spouses. Similarly, another study [29] found an association between lower socioeconomic status and higher depression and suggested that low and unstable socioeconomic status increases economic stress and fosters negative self-concepts, consequently causing depression. Further, existing studies have shown that individuals without spouses can easily experience depression as a result of experiencing difficulties and helplessness when attempting to perform tasks with which spouses usually assist [30]. The above findings are supported by the 2016 Epidemiological Survey of Mental Illness [24], which reported that the annual prevalence of depression was higher in groups with “bottom” incomes than in those with middle (2.7%) and high (1.1%) incomes, and that it was also higher among unmarried individuals (2.3%) and individuals who were divorced, separated, or separated by death (3.1%) than among married individuals (1.0%). Therefore, it is necessary to consider socioeconomic status and spouse status when seeking to develop approaches for reducing depression.

Another previous study [31] reported a relationship between drinking and depression, suggesting that having a drinking problem predicts high depression, and that heavy drinking or alcoholism creates a high probability of depression. In the present study, we found that frequency of drinking affects depression regardless of practice in physical activity; thus, it is expected that interventions that aim to address unhealthy drinking behaviors can contribute to lowering depression.

Subjective health represents self-awareness of one’s health, and better subjective health has been found to be associated with a lower likelihood of depression [32]; this accords with the present findings. As subjective health is closely related to negative mental health, such as feelings of depression and a sense of social isolation [33], efforts to improve subjective health could represent another important means of reducing depression.

The finding of this study that lower perceived stress is associated with lower depression is consistent with previous findings that long-term stress increases the risk of depression [34]. Stress has a negative impact on depression, and interventions designed to reduce stress are essential for lowering depression and improving depressive symptoms.

In this study, individuals who perceived themselves as being of average weight showed lower depression than those who perceived themselves as being obese. Similarly, previous studies [35] have reported an association between obesity and depression; in particular, Korean culture pressures people to be thin can cause psychological problems among people [36]. Thus, depression intervention strategies should feature an understanding of such cultural characteristics and, for those determined through objective measurement to actually be obese, measures for addressing associated health problems should be included. In addition, in the case of an incorrect perception of obesity, a strategy to foster a positive body image and accurate recognition of one’s own body status is required.

As experience of controlling one’s own weight can have a positive effect on depression by providing a sense of self-control and self-esteem [37], we expected that, in the no-aerobic physical activity group, BMI would have a significant impact on depression when compared to the aerobic physical activity group.

The present study found that, in the aerobic physical activity group, individuals in their 30s were more likely to have depression than those aged 65 or older. A previous study reported that depressive symptoms increase during one’s 30s, decrease in middle age, and then rise sharply once the individual exceeds 80 years of age [38]. Further, along with the 20s, the 30s are associated with a high risk of suicide and suicide attempts [39]. Compounding this situation is the fact that, today, the younger generation uses a variety of social media, and it has been reported that the use of such social media can unintentionally increase depression and anxiety [40].

Depression is deeply related to economic status; the youth unemployment rate was higher than any age group [41], and youth unemployment has a negative impact on mental health as well as physical health [42]. Economic instability is a major cause of financial instability and unemployment which, according to a previous study [43], is contributing to the continuously rising prevalence of depression among young individuals. In this study, the aerobic physical activity group contained more people in their 30s, which may explain why, in this group, age was a significant factor for depression. Thus, as age is a factor influencing depression, when seeking to address depression, it is necessary to understand the characteristics that uniquely affect each age group.

In this study, a lower education level was found to be associated with higher depression; this is similar to previous findings [44]. Low educational attainment has been found to increase the risk of exposure to incidents such as injuries and psychological trauma, which can be causes of depression [45]. However, further studies are required to verify this effect because there are few existing studies concerning whether the influence of education level on depression status differs across different levels of aerobic activity.

One of the strengths of this study is that it utilized data from a large population sample. The participants in the KNHANES are a nationally representative sample of civilians in South Korea, and it is possible to generalize results by conducting the analysis according to the guidelines of the KCDC [21].

This study has some limitations. First, as a cross-sectional study, the possibility of reverse causality between depression and physical activity cannot be completely excluded, and causal inferences cannot be made. Thus, it will be necessary in the future to increase the strength of evidence through longitudinal research. Second, although it is possible to extend the interpretation to Korean adults, it would be difficult to extend the interpretation of the results to other countries with cultural differences. Finally, variable extraction was limited because data collected by the government, and not directly collected by the researcher, was used for research.

## 5. Conclusions

Depression is a representative mental health disease that negatively affects not only an individual’s life but also their family and community. Therefore, effectively mediating depression is not an individual problem, but a global task. Aerobic physical activity has previously identified as a method of intervention that has a positive effect on depression. However, studies have applied each type of physical activity as a separate intervention or simply confirmed the influencing factors. The greatest significance of this study is that it integrates them, identifies the influencing factors, and conducts a comparative analysis. Moreover, considering the importance of depression in improving public health, this study is notably population-based. The results of this study, using representative national data in establishing strategies to mediate depression in adults in the future, are valuable as basic data for establishing public health strategies. Considering the differences in the influencing factors revealed through this study, we propose a longitudinal study to verify the effectiveness of an appropriate strategy to mediate depression for each target group.

## Figures and Tables

**Table 1:** Comparison of the no aerobic physical activity and aerobic physical activity groups regarding sociodemographic characteristics (*n* = 12,891).

Characteristics		Total *n* (Weight %)	No Aerobic Physical Activity *n* (Weighted %)	Aerobic Physical Activity *n* (Weighted %)	χ^2^ (*p*)
Gender	Men	5702 (50.2)	2986 (47.6)	2716 (53.2)	40.513 (<0.001)
	Women	7189 (49.8)	4162 (52.4)	3027 (46.8)	
Age (years)	30–49	5443 (50.0)	2731 (45.8)	2712 (54.7)	170.662 (<0.001)
	50–64	4098 (32.7)	2265 (33.2)	1833 (32.2)	
	≥65	3350 (17.3)	2152 (21.0)	1198 (13.1)	
Family income	Upper	3771 (31.5)	1844 (27.8)	1927 (35.7)	150.693 (<0.001)
	Middle	6896 (55.1)	3852 (56.1)	3044 (54.1)	
	Lower	2188 (13.4)	1426 (16.1)	762 (10.2)	
Education level	≤Middle school	4156 (26.1)	2744 (32.1)	1412 (19.2)	293.818 (<0.001)
	High school	3920 (32.6)	2064 (31.2)	1856 (34.2)	
	≥College	4784 (41.3)	2319 (36.7)	2465 (46.6)	
Number of household members	1	1346 (8.6)	819 (9.2)	527 (7.9)	33.348 (<0.001)
	2	3908 (25.8)	2281 (27.5)	1627 (23.9)	
	≥3	7637 (65.6)	4048 (63.3)	3589 (68.2)	
Housing type	Detached house	4248 (31.3)	2552 (33.4)	1696 (29.0)	32.649 (<0.001)
	Apartment	7122 (55.7)	3744 (53.5)	3378 (58.1)	
	Other	1521 (13.0)	852 (13.1)	669 (12.9)	
Marital status	Living with spouse	10,303 (87.7)	5626 (85.4)	4677 (90.4)	68.653 (<0.001)
	Other	1740 (12.3)	1135 (14.6)	605 (9.6)	

**Table 2:** Comparison of the no aerobic physical activity and aerobic physical activity groups in terms of physical and psychological factors (*n* = 12,891).

Characteristics		Total *n* (Weight %)/M (SE)	No Aerobic Physical Activity *n* (Weight %)/M (SE)	Aerobic Physical Activity *n* (Weight %)/M (SE)	χ^2^/t (*p*)
Blood pressure	Normal	5346 (43.7)	2800 (41.4)	2546 (46.3)	59.253 (<0.001)
	Prehypertension	3145 (25.8)	1677 (25.2)	1468 (26.4)	
	Hypertension	4364 (30.5)	2656 (33.4)	1708 (27.3)	
Body weight	Underweight	381 (3.4)	231 (4.0)	150 (2.7)	13.852 (0.001)
	Normal	6844 (61.2)	3678 (60.4)	3166 (62.1)	
	Obese	3922 (35.4)	2168 (35.6)	1754 (35.2)	
Diabetes status	Normal	7301 (61.9)	3881 (59.9)	3420 (64.2)	27.945 (<0.001)
	Impaired fasting glucose	3148 (26.7)	1773 (27.5)	1375 (25.6)	
	Diabetes	1565 (11.4)	961 (12.6)	604 (10.2)	
Hypercholesterolemia status	No hypercholesterolemia	9423 (80.0)	5119 (79.0)	4304 (81.1)	8.583 (0.004)
	Hypercholesterolemia	2604 (20.0)	1505 (21.0)	1099 (18.9)	
Weight change	Increased	2716 (22.1)	1494 (22.2)	1222 (22.0)	0.045 (0.975)
	Decreased	1519 (12.1)	841 (12.1)	678 (12.2)	
	No change	8573 (65.8)	4752 (65.7)	3821 (65.8)	
Weight control	Made efforts to reduce	5108 (41.0)	2550 (36.7)	2558 (45.9)	292.777 (<0.001)
	Made efforts to Maintain	2412 (18.8)	1165 (16.4)	1247 (21.6)	
	Made efforts to increase	605 (4.7)	344 (4.8)	261 (4.4)	
	Made no efforts	4700 (35.5)	3041 (42.1)	1659 (28.1)	
Drinking	No	1463 (9.1)	908 (10.3)	555 (7.7)	26.454 (<0.001)
	Yes	11,360 (90.9)	6190 (89.7)	5170 (92.3)	
Frequency of drinking	≤1/month	5618 (46.0)	3185 (47.7)	2433 (44.2)	19.342 (<0.001)
	2–4/month	2723 (25.5)	1386 (24.0)	1337 (27.2)	
	≥2/week	3018 (28.5)	1618 (28.3)	1400 (28.6)	
Alcohol consumption per session (glasses)	1–2	3653 (35.1)	1956 (35.4)	1697 (34.8)	0.633 (0.719)
	3–6	3350 (36.7)	1780 (36.8)	1570 (36.6)	
	≥7	2213 (28.2)	1140 (27.8)	1073 (28.6)	
Smoking	No	1561 (54.7)	4230 (55.2)	3331 (54.2)	1.306 (0.232)
	Yes	5254 (45.3)	2862 (44.8)	2392 (45.8)	
Subjective health	Good	3865 (30.9)	1849 (26.3)	2016 (36.2)	178.686 (<0.001)
	Normal	7021 (54.7)	4005 (56.9)	3016 (52.3)	
	Bad	2004 (14.4)	1293 (16.8)	711 (11.5)	
Subjective body weight	Thin	1897 (14.6)	1122 (15.9)	775 (13.1)	20.499 (<0.001)
	Normal	5288 (40.4)	2921 (40.1)	2367 (40.8)	
	Obese	5636 (45.0)	3054 (44.0)	2582 (46.1)	
Perceived stress	Low	9933 (76.3)	5448 (75.6)	4485 (77.1)	3.938 (0.040)
	High	2878 (23.7)	1641 (24.4)	1237 (22.9)	
Depression		2.06 (0.031)	2.11 (0.042)	2.0 (0.035)	2.309 (0.022)

M: mean; SE: standard error.

**Table 3:** Factors associated with depression (*n* = 12,891).

Characteristics		No Aerobic Physical Activity			Aerobic Physical Activity		
		β	t	*p*	β	t	*p*
Gender	Men	−0.629	−7.390	<0.001	−0.530	−7.020	<0.001
	Women	1.0			1.0		
Age (years)	30–49	−0.170	−1.264	0.207	0.360	2.964	0.003
	50–64	−0.217	−1.986	0.048	0.060	0.550	0.582
	≥65	1.0			1.0		
Family income	Upper	−0.362	−2.631	0.009	−0.418	−2.697	0.007
	Middle	−0.141	−1.076	0.283	−0.293	−2.045	0.042
	Lower	1.0			1.0		
Education level	≤Middle school	−0.076	−0.714	0.476	0.269	2.516	0.012
	High school	0.035	0.447	0.655	0.260	3.434	0.001
	≥College	1.0			1.0		
Housing type	Detached house	0.167	1.339	0.182	0.257	2.560	0.011
	Apartment	0.095	0.787	0.432	0.183	1.917	0.056
	Other	1.0			1.0		
Marital status	Living with spouse	−0.369	−2.215	0.027	−0.745	−3.307	0.001
	Other	1.0			1.0		
Blood pressure	Normal	0.209	2.012	0.045	0.347	3.430	0.001
	Prehypertension	0.198	1.966	0.050	0.165	1.721	0.086
	Hypertension	1.0			1.0		
Body weight	Underweight	1.166	4.498	<0.001	0.408	1.809	0.071
	Normal	0.409	4.815	<0.001	0.289	3.182	0.002
	Obese	1.0			1.0		
Hypercholesterolemia status	No hypercholesterolemia	−0.078	−0.788	0.432	0.209	2.202	0.028
	Hypercholesterolemia	1.0			1.0		
Frequency of drinking	≤1/month	−0.443	−4.703	<0.001	−0.300	−3.701	<0.001
	2–4/month	−0.402	−4.107	<0.001	−0.297	−3.127	0.002
	≥2/week	1.0					
Subjective health	Good	−2.001	−15.253	<0.001	−2.341	−13.627	<0.001
	Normal	−1.286	−10.458	<0.001	−1.774	−10.222	<0.001
	Bad	1.0			1.0		
Subjective body weight	Thin	−0.320	−2.328	0.021	−0.134	−1.010	0.313
	Normal	−0.340	−3.909	<0.001	−0.323	−3.871	<0.001
	Obese	1.0			1.0		
Stress	Low	−2.213	−20.730	<0.001	−2.210	−20.964	<0.001
	High	1.0			1.0		
R^2^/F/*p*		R^2^ = 0.213, F = 41.475, *p* < 0.001			R^2^ = 0.246, F = 40.190, *p* < 0.001		

## Data Availability

KNHANES data are publicly accessible. The data can be accessed and downloaded from the KNHANES homepage (URL: https://knhanes.kdca.go.kr/knhanes/eng/index.do, accessed on 7 March 2021).
